# Precision Medicine for Metastatic Colorectal Cancer: Where Do We Stand?

**DOI:** 10.3390/cancers16223870

**Published:** 2024-11-19

**Authors:** Patrick W. Underwood, Timothy M. Pawlik

**Affiliations:** Department of Surgery, The Ohio State University Wexner Medical Center, 395 W. 12th Ave., Suite 670, Columbus, OH 43210, USA; patrick.underwood@osumc.edu

**Keywords:** targeted therapy, colon cancer, molecular profiling, personalized medicine

## Abstract

Metastatic colorectal cancer is a leading cause of cancer-related death across the world. The treatment paradigm has shifted away from systemic chemotherapy alone to include targeted therapy and immunotherapy. The past two decades have been characterized by increased investigation into molecular profiling of colorectal cancer. These molecular profiles help physicians to better understand colorectal cancer biology among patients with metastatic disease. Additionally, improved data on genetic pathways allow for specific therapies to be targeted at the underlying molecular profile. Investigation of the EGFR, VEGF, HER2, and other pathways, as well as deficient mismatch repair, has led to the development of multiple targeted therapies that are now utilized in the National Comprehensive Cancer Network guidelines for colon and rectal cancer. While these new therapies have contributed to improved survival for metastatic colorectal cancer, long-term survival remains poor. Additional investigation to understand resistance to targeted therapy and development of new targeted therapy is necessary. New therapies are under development and are being tested in the preclinical and clinical settings. The aim of this review is to provide a comprehensive evaluation of molecular profiling, currently available therapies, and ongoing obstacles in the field of colorectal cancer.

## Simple Summary

The treatment paradigm for metastatic colorectal cancer has significantly evolved over the past two decades. Investigators have focused on molecular profiling of tumors and developing targeted therapies. In turn, several first-line targeted therapies have been approved for use in metastatic colorectal cancer with a subsequent improvement in survival outcomes. Nevertheless, durable response to therapy and long-term survival remains elusive for patients with metastatic colorectal cancer. Continued development of new targeted therapies and investigation of combined targeted therapies are needed.

## 1. Introduction

Colorectal cancer (CRC) is the fourth most commonly diagnosed cancer in the United States, with an estimated 152,810 cases expected in 2024 [1]. CRC is the second leading cause of cancer-related death, with 53,010 new deaths expected in 2024. The incidence and mortality of CRC have decreased over the past decade with increased screening [2,3,4]. Nevertheless, 25–50% of patients will present with or develop liver metastasis during the course of their disease [5,6]. Approximately 13–25% of patients will develop metachronous liver metastasis after curative-intent resection of the primary tumor. The median survival for patients with metastatic CRC (mCRC) is 32–40 months [7,8]. The expected 5-year survival is 35–65% [9].

Over the past decade, treatment for colorectal liver metastasis has greatly improved. Treatment options include resection, liver-directed therapies, targeted therapy, immunotherapy, and systemic chemotherapy. The optimal approach to treatment requires a multidisciplinary team that involves surgeons, medical oncologists, radiation oncologists, diagnostic radiology, and interventional radiology [10]. Previous data suggest that a multidisciplinary team can improve survival for patients with mCRC [11,12]. Current efforts to improve survival for mCRC are focused on personalized approaches with targeted therapies toward genetic mutations [13]. This review focuses on currently approved targeted therapies for colorectal liver metastasis (CRLM), ongoing obstacles in treatment, and future directions. 

## 2. Methods

A review of the literature was performed using medline/PubMed. The search terms “colorectal cancer”, “targeted therapies”, “personalized medicine”, “immunotherapy”, and “colorectal liver metastasis” were used. The search ended on 25 September 2024. P.W.U. performed the initial literature review, and the final selection was made by T.M.P. The selection criteria focused on clinical trials leading to currently approved therapies for CRLM, promising new therapies, and other potential targetable mutations.

## 3. Genomic and Molecular Profiling in Colorectal Cancer

Systemic chemotherapy was the mainstay of treatment for mCRC, but genetic profiling has identified several pathways in the pathogenesis of CRC with actionable targets. Current U.S. Food and Drug Administration (FDA)-approved targeted therapies involve the epidermal growth factor receptor (EGFR), Vascular Endothelial Growth Factor (VEGF), HER2, and tyrosine receptor kinase fusion pathways [14]. Additionally, checkpoint inhibition is effective, and immunotherapy is approved for patients with microsatellite instability. Table 1 displays the targetable pathways, prevalence, and available therapies for patients with mCRC. With currently available therapies, multidisciplinary teams treating patients with mCRC require rapid information on RAS mutations, BRAFV600 mutations, and microsatellite instability [15]. The National Comprehensive Cancer Network (NCCN) guidelines recommend testing for KRAS/NRAS and BRAF mutations, HER2 amplification, and microsatellite instability. While genetic alterations may be tested individually, NCCN guidelines recommend Next Generation Sequencing (NGS) that can find additional, rarer mutations. The genetic profiles of primary tumors and metastatic lesions are generally similar [16,17]. Overall, targeted therapies are ineffective when there are downstream mutations from the target. Therefore, these genomic and molecular variants are critical to determine optimal therapy for patients with mCRC.

An international consortium defined four different consensus molecular subtypes (CMS) to classify CRC [18]. The molecular subtypes are displayed in Table 2. These subtypes offer opportunities for classification and targeted therapy. Germline mutations exist in about 6–10% of patients with CRC [19,20]. Lynch syndrome pathogenic variants (MLH1, MSH2, MSH6, and PMS2) are the most common. Other high penetrance mutations include APC, biallelic MUTYH, BRCA1/2, PALB2, CDKN2A, and TP53 [20]. NCCN guidelines recommend genetic testing for anyone with a personal or family history of a known pathogenic variant, personal or family history of >10 adenomatous polyps ≥2 hamartomatous polyps, or ≥5 serrated polyps proximal, personal or family history of Lynch syndrome-related cancer.

## 4. Epidermal Growth Factor Pathway Signaling Inhibitors

**Figure 1:**
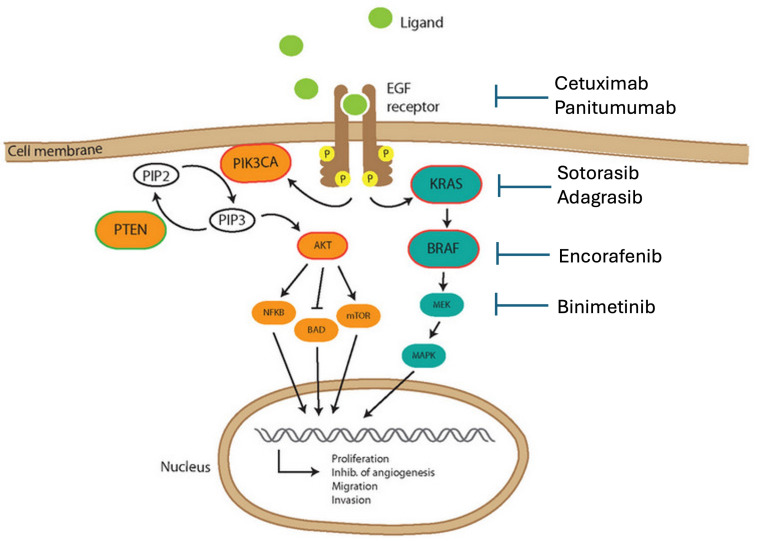
The EGFR pathway and downstream targets for therapy. Figure adapted from Berg et al. [22].

The epidermal growth factor receptor (EGFR) is a transmembrane receptor in a family of four receptor tyrosine kinases. After ligand binding, the receptor forms a dimer that activates the downstream intracellular pathway, including RAS, RAF, MEK, and ERK. This downstream signaling leads to cell proliferation. Figure 1 displays the EGFR pathway. The EGFR pathway has been implicated in the carcinogenesis of multiple cancers, including lung, colorectal, squamous cell carcinoma of the head and neck, and pancreatic cancer [21]. Multiple targeted therapies have been developed and approved by the FDA for use in CRC.

### 4.1. EGFR Targeted Therapy

Cetuximab was the first FDA-approved targeted therapy for use in mCRC in 2004. Cetuximab approval came after the results of the BOND trial [23]. In this trial, patients who had disease progression on irinotecan-based regimens were randomly assigned to cetuximab monotherapy versus cetuximab and irinotecan. Patients in the combination therapy group experienced improved progression-free survival (4.1 versus 1.5 months). The EPIC trial was a multicenter, phase III, randomized controlled trial (RCT) that reported improved progression-free survival (PFS) and objective response rate (ORR) among patients treated with cetuximab and irinotecan compared with irinotecan alone as second-line therapy [24]. Patients treated with cexutimab and irinotecan experienced 4.0 versus 2.6 months median PFS.

Further research has demonstrated that the benefits of cetuximab therapy were confined to patients who had KRAS wild-type (wt) CRC [25,26,27]. RAS is activated by EGFR, which would explain the poor response to EGFR therapy in KRAS mutant CRC. The CRYSTAL study confirmed the value of cetuximab among patients with KRAS wt tumors who were treated with cetuximab plus FOLFIRI versus FOLFIRI alone [28]. This study also noted that BRAF mutation was an independent predictor of poor prognosis. As shown in Figure 1, BRAF is a downstream protein from EGFR and KRAS. The TAILOR trial, a multicenter, phase III RCT, investigated cetuximab plus FOLFOX-4 versus FOLFOX-4 alone as a first-line therapy for RAS (KRAS and NRAS) wt tumors [29]. The investigators reported that the addition of cetuximab to FOLFOX-4 significantly improved survival. This study led to NCCN guidelines recommending cetuximab in addition to systemic chemotherapy as first-line therapy for RAS wt tumors.

Cetuximab has been studied in the perioperative setting for patients with resectable mCRC. A recent multicenter, phase III RCT examined the use of systemic chemotherapy with or without cetuximab before and after liver resection [30]. In this patient population, there was a significant decrease in median OS for patients in the cetuximab group from 81.0 months to 55.4 months. Cetuximab, therefore, should be avoided in the perioperative setting. 

Panitumumab, another EGFR antagonist, is a human monoclonal antibody that received FDA approval in 2007. Similar to cetuximab, initial trials noted benefits in PFS for patients with treatment-refractory mCRC [31]. A multicenter RCT in Belgium noted that panitumumab plus FOLFIRI was superior to FOLFIRI alone as second-line therapy for mCRC with RAS wt in PFS (6.4 versus 3.7 months) [32]. The PRIME trial, a multinational, multicenter phase III RCT, evaluated the efficacy of FOLFOX-4 with or without panitumumab [33]. Panitumumab with FOLFOX-4 had superior PFS (10.0 versus 8.6 months, *p* = 0.01). The ASPECCT and WJOG 6510G trials both demonstrated non-inferiority of panitumumab compared with cetuximab when combined with systemic therapy for KRAS wt mCRC [34,35].

The colon has different embryologic origins. The left side of the colon is derived as part of the hindgut with arterial supply from the inferior mesenteric artery, and the right side of the colon is derived as part of the midgut with arterial supply from the superior mesenteric artery. Multiple studies have noted that targeting EGFR has a significant effect in improving PFS in left-, but not right-, sided mCRC [36,37]. This observation has led to NCCN guideline recommendations for panitumumab or cetuximab plus systemic therapy in patients with RAS wt left-sided mCRC.

### 4.2. RAS

RAS is a family of proteins in the EGFR pathway. RAS is a GTPase involved in cellular signal transduction. When activated, RAS promotes cell growth, differentiation, and survival. RAS variants have been implicated in many cancers [38]. KRAS mutations can be present in greater than 50% of CRC [39]. Multiple agents have been developed to target KRAS, and two have been approved for clinical use [40]. Sotorasib was developed to inhibit KRAS G12C, which is present in about 4% of CRC [39]. Initial trials investigating sotorasib monotherapy for patients with KRAS G12C mutations, including the CodeBreaK100 trial, demonstrated minimal efficacy [41]. A recent trial published in the New England Journal of Medicine evaluated sotorasib in chemorefractory mCRC [42]. The 3 arms of the multicenter phase III RCT were sotorasib 960 mg daily plus panitumumab, sotorasib 240 mg daily plus panitumumab, or the investigator’s choice of trifluridine-tipiracil or regorafenib. The investigators reported improved PFS of 5.6 months, 3.9 months, and 2.2 months in the high-dose sotorasib, low-dose sotorasib, and standard-care groups, respectively. The CodeBreaK 301 trial is underway, which is investigating sotorasib and panitumumab with FOLFIRI as first-line therapy in KRAS G12C mutant mCRC [43].

Adagrasib is another inhibitor developed to target mutant KRAS G12C protein. A phase I–II clinical trial, KRYSTAL-1, investigated adagrasib monotherapy versus adagrasib with cetuximab in chemorefractory mCRC [44]. The monotherapy group had a PFS of 5.6 months compared with 6.9 months in the combination therapy group. The results of this trial led to FDA approval of adagrasib in combination with cetuximab for patients with mutant KRAS G12C previously treated with systemic chemotherapy. Current NCCN guidelines recommend either sotorasib or adagrasib plus cetuximab or panitumumab for mutant KRAS G12C mCRC for patients previously treated with systemic FOLFOX/CAPEOX.

### 4.3. BRAF/MEK

BRAF is a proto-oncogene that encodes the B-RAF protein downstream from EGFR. It is a growth signal transduction protein that regulates the MAPK pathway and is involved in cell growth and division. Approximately 8–12% of patients with mCRC have a BRAF V600E mutation [45]. Encorafenib was initially investigated as a selective inhibitor of RAF kinase with clinical applicability in melanoma [46]. Approximately 8–12% of patients with mCRC have a BRAF V600E mutation [45]. This finding led investigators to study the use of encorafenib in mCRC. Investigators evaluated combination-targeted therapies in an effort to improve response to therapy and overcome the development of drug resistance [47]. MEK is another protein kinase in the MAPK pathway that is involved in cell growth and division. Binimetinib was developed as an inhibitor of MEK [48].

The BEACON trial was a multinational, multicenter, phase III RCT that investigated the use of encorafenib and binimetinib in patients with mCRC and BRAF V600E mutations previously treated with standard chemotherapy [49]. BRAF V600E mutations were confirmed by a central laboratory as part of molecular prescreening. The trial enrolled patients into three arms: encorafenib, binimetinib, and cetuximab; encorafenib and cetuximab; or investigator choice of cetuximab and systemic chemotherapy (irinotecan or FOLFIRI). An updated analysis of this trial demonstrated improved overall survival (OS), objective response rate (ORR), and PFS of the encorafenib and cetuximab groups compared with the control group [50]. The addition of binimetinib to encorafenib and cetuximab did not improve OS versus encorafenib and cetuximab. This may be due to the rarity of MEK mutations (1%) [51]. Therefore, the most recent NCCN guidelines recommend encorafenib plus cetuximab for second-line treatment in mCRC.

## 5. Vascular Endothelial Growth Factor Inhibitors

**Figure 2:**
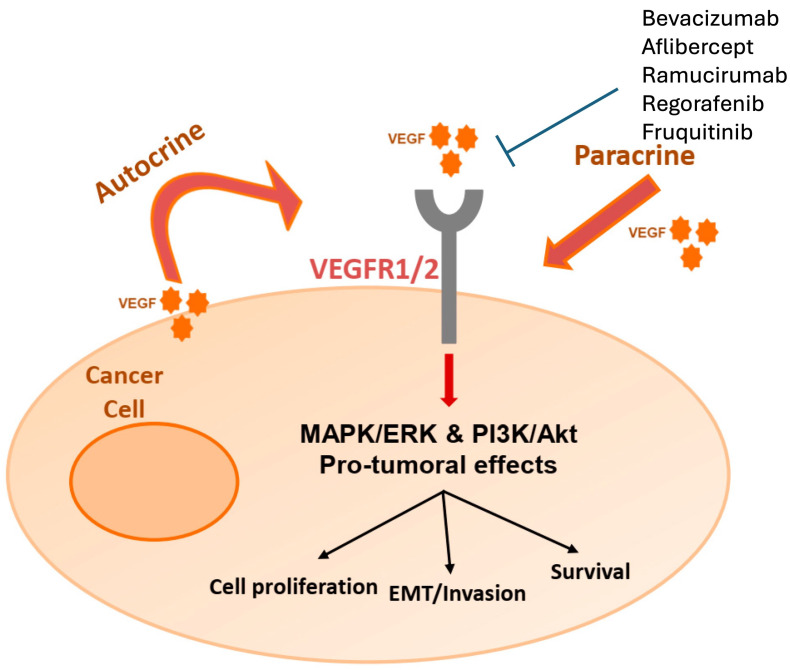
The VEGF pathway and its effect on tumorigenesis. Adapted from Ntellas et al. [54].

The vascular endothelial growth factor (VEGF) pathway is another successfully targeted pathway for mCRC. Figure 2 displays the VEGF pathway. VEGF is a protein produced by a variety of cells to stimulate angiogenesis [52]. When functioning normally, hypoxic conditions cause the release of VEGF, which then helps to create new blood vessels. Cancers that express VEGF can develop new blood supply to help tumors grow and metastasize. Investigators have developed several therapies to target VEGF and inhibit tumor growth. VEGF inhibition alone is not cytotoxic to tumor cells. VEGF inhibitors are felt to be chemosensitizers to promote tumor cell death [53]. VEGF inhibition is also studied with immunotherapy, which is discussed further below.

Bevacizumab is the first FDA-approved VEGF inhibitor for use in mCRC. It is a humanized monoclonal antibody that inhibits VEGF-A isoforms. It was studied as first-line therapy in a multicenter RCT [55]. Patients were randomized to irinotecan, fluoruracil, and leucovorin with or without bevacizumab. Median survival was 20.3 months in the bevacizumab group compared with 15.6 months in the control group. PFS was 10.6 versus 6.2 months in the bevacizumab and control groups, respectively. A 2 × 2 RCT was then performed comparing XELOX versus FOLFOX-4 with bevacizumab or placebo as first-line therapy for mCRC [56]. The investigators noted that the addition of bevacizumab to either oxaliplatin-based regimen improved median PFS from 8.0 months to 9.4 months and median OS from 19.9 months to 21.3 months. Another multicenter, phase III RCT investigated FOLFOX-4 with and without bevacizumab for patients previously treated with CRC with fluoropyrimidine and irinotecan [57]. The study investigators noted a median survival of 12.9 versus 10.8 months in the FOLFOX-4 plus bevacizumab versus the FOLFOX-4 alone group.

The utility of bevacizumab has been studied in several other settings. The ML18147 trial studied continuing bevacizumab in second-line therapy after progression with first-line chemotherapy, including bevacizumab [58]. The results of the multicenter, phase III RCT found that maintenance of bevacizumab with second-line therapy improved OS compared to second-line therapy without bevacizumab. The CAIRO3 study examined the maintenance of bevacizumab and capecitabine for patients previously treated with six cycles of CAPEOX and bevacizumab [59]. The results of this multicenter RCT noted that PFS was improved in the maintenance bevacizumab/capecitabine group compared with observation. Bevacizumab has also been studied as adjuvant therapy for patients with resected stage III CRC in the S-AVANT trial [60]. This phase III RCT reported no benefit to adjuvant bevacizumab after curative resection.

Several other VEGF inhibitors have been studied more recently. Aflibercept is considered a “VEGF trap” as it binds to circulating VEGF. Aflibercept was studied in the phase II AFFIRM trial [61]. Patients were randomized to mFOLFOX-6 with or without aflibercept. The trial noted no difference in PFS and higher levels of toxicity. Ramucirumab is a humanized monoclonal antibody that targets VEGF Receptor-2 (VEGFR-2). Ramucirumab was studied in the multicenter, phase III RAISE trial as second-line therapy [62]. Patients with progression of mCRC were randomized to FOLFIRI with or without ramucirumab. Median OS was improved at 13.3 months in the ramucirumab group versus 11.7 months in the control group.

Regorafenib was developed as a multikinase inhibitor that exhibits effects on VEGFR, platelet-derived growth factor receptor, fibroblast growth factor receptor, and BRAF [63]. Its use in colon cancer has been studied in multiple trials with mixed results. The CORRECT trial was a multicenter, phase III RCT comparing regorafenib to best supportive care in patients with mCRC refractory to standard treatment [64]. Patients treated with Regorafenib had 6.5 months median OS versus 5.0 months in the placebo group. The CONCUR trial was a similar multicenter, phase 3 trial in Asian patients comparing regorafenib to placebo [65]. This trial supported the CORRECT trial findings with a median OS of 8.8 months versus 6.3 months in the placebo group. Regorafenib was studied as first-line therapy in a multicenter, phase II RCT [66]. The trial compared mFOLFOX-6 with regorafenib to the historical control of FOLFOX-6 alone. Unfortunately, the addition of regorafenib did not improve OS. 

Fruquintinib is another kinase inhibitor that inhibits VEGF-induced phosphorylation of the VEGFR. Fruquintinib was studied in mCRC in the FRESCO and FRESCO-2 trials. The FRESCO trial compared Fruquintinib versus placebo as third-line therapy in patients with mCRC [67]. The trial was a multicenter, phase III RCT in China. Patients treated with fruquintinib had a median OS of 9.3 versus 6.6 months in the placebo group. The FRESCO-2 trial further supported these findings [68]. The multicenter, phase III RCT compared fruquintinib to placebo in Japanese patients with mCRC. The median OS was 7.4 versus 4.8 months in the fruquintinib and control groups, respectively. Fruquintinib received FDA approval for treatment of refractory mCRC in 2023. 

Taken together, these therapies and trials demonstrate VEGF as a viable target for therapy. NCCN guidelines recommend the use of bevacizumab with FOLFOX or CAPEOX for mCRC. Aflibercept and ramucirumab have indications for mCRC as second-line therapy. Fruquintinib and regorafenib are reserved for treatment refractory mCRC that has progressed through multiple lines of therapy.

## 6. HER2 Inhibitors

**Figure 3:**
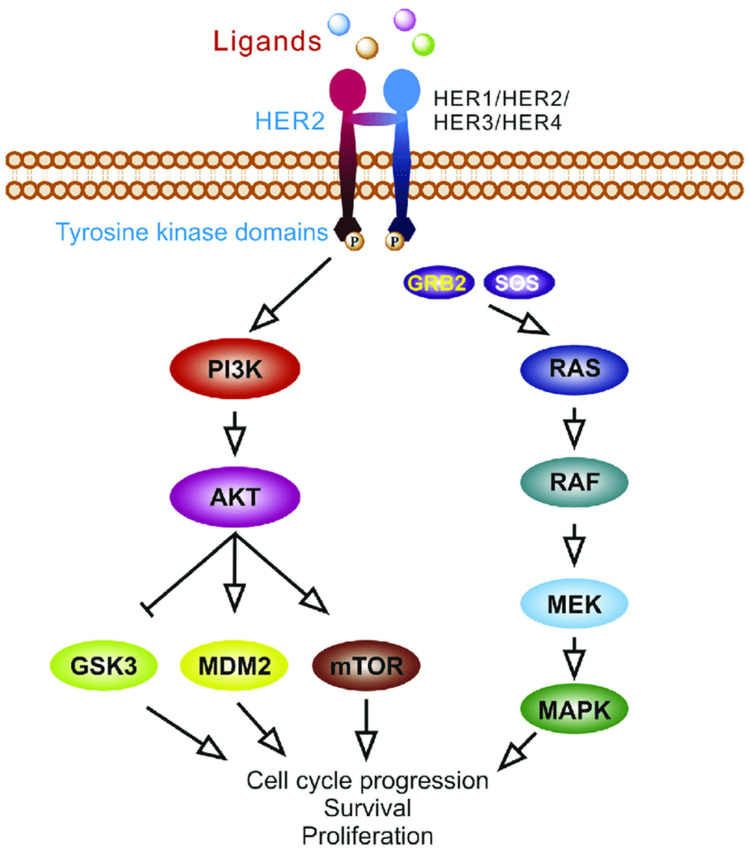
The HER2 pathway and its effect on tumorigenesis. Adapted from Feng et al. [71].

Human epidermal growth factor receptor-2 (HER2), also known as ERRB-2, is similar to EGFR as it is part of the ERBB family of proteins (Figure 3). ERRB-2 has similar downstream pathways involving RAS/RAF/MEK. Inhibition of HER2 has been well studied in breast cancer, in which HER2 overexpression is noted in about 20% of cases [69]. In CRC, HER2 overexpression is only present in about 3–5% of cases [70]. Nevertheless, many of the same treatments used in breast cancer can be used to target mCRC.

Trastuzumab has long been approved for use in HER2-positive breast cancer. Trastuzumab is a monoclonal antibody that binds to the HER2 receptor and inhibits cellular proliferation. Trastuzumab was studied in combination with pertuzumab, another HER2 inhibitor, in the phase II MyPathway trial [72]. The trial demonstrated an ORR of 32% in previously treated patients with mCRC. The TAPUR study was a similar phase II trial that studied trastuzumab in combination with pertuzumab and noted an ORR of 25% [73]. Trastuzumab has also been studied in combination with lapatinib. Lapatinib is a tyrosine kinase inhibitor that targets both HER2 and EGFR. Together, pertuzumab and lapatinib were studied in the HERACLES trial [74]. This multicenter, phase II trial reported a 30% ORR with good overall tolerance. The combination of trastuzumab and tucatinib, another HER2 inhibitor, was studied in the phase II MOUNTAINEER trial for patients with chemorefractory mCRC [75]. Of the 84 patients receiving the combination, 38.1% had an objective response.

Trastuzumab deruxtecan (T-DXd) was developed as a HER2 inhibitor and topoisomerase inhibitor. It was initially studied in the phase II DESTINY-CRC01 trial [76]. The trial investigated T-DXd in patients with chemorefractory HER2+ KRAS wt mCRC to at least 2 previous regimens. The trial reported an ORR of 45.3% with PFS of 6.9 months and OS of 15.5 months. The DESTINY-CRC02 trial included both KRAS wt and mutant disease for individuals with previously treated mCRC [77]. The investigators reported an ORR of 37.8% in the 5.4 mg/kg group regardless of KRAS status. The results of these trials led to NCCN guideline recommendations for Trastuzumab with either pertuzumab, lapatinib, or tucatinib for KRAS wt mCRC previously treated with FOLFOX/CAPEOX or T-DXd alone for KRAS wt or mutant mCRC previously treated with FOLFOX/CAPEOX.

## 7. Neurotrophic Receptor Tyrosine Kinase Fusion

Neurotrophic receptor tyrosine kinases (NTRK) are a family of genes that encode tropomyosin receptor kinases (TRK). These protein receptors are involved in neural cell development. NTRK gene fusions lead to TRK fusion proteins with activation of the downstream pathways leading to oncogenesis [78]. These gene fusions are extremely rare and represent only about 0.7% of CRC [79]. Nevertheless, when identified, they represent a target for FDA-approved therapy.

Entrectinib is a selective tyrosine kinase inhibitor used in the treatment of NTRK fusion-positive solid tumors. Due to the rarity of NTRK tumors, its efficacy has been studied in clinical trials of multiple solid organ tumor types. The results of three clinical trials (ALKA-372-001, STARTRK-1, and STARTRK-2) were published together [80]. Of the 54 patients in the studies, only 4 (7%) were treated for mCRC. Of the 54 patients, 31 (57%) experienced an objective response (OR), and 4 (7%) experienced a complete response (CR). Larotrectinib is a tropomyosin kinase receptor inhibitor developed to target NTRK fusion-positive tumors. Similar to entrectinib, its use was studied in three clinical trials with a small number of patients in multiple solid tumor types [81]. In this study, 4/55 (7%) were treated for CRC. The ORR was 75%. Among patients with a response, 71% had an ongoing response at one year. Repotrectinib is the third FDA-approved tropomyosin kinase receptor inhibitor. Repotrectinib use was studied in the TRIDENT-1 study [82]. The results of this trial were reported in patients with non-small cell lung cancer and NTRK fusion-positive tumors. Most patients had been previously treated with a different TRK inhibitor. The investigators observed a 79% ORR and a median duration of response of 34.1 months. The NCCN guidelines recommend the use of entrectinib, larotrectinib, or repotrectinib for patients with mCRC and NTRK fusion-positive tumors.

## 8. RET Fusion

RET proto-oncogene encodes a cell surface receptor tyrosine kinase long known to be involved in tumorigenesis. Selpercatinib was developed as a tyrosine kinase inhibitor to target RET fusion-positive tumors. Approximately 0.2% of CRCs are RET fusion-positive [83]. It was studied in the LIBRETTO-001 trial [84]. The reported results included 45 patients with RET fusion-positive tumors, of which 10 (22%) had mCRC. The ORR was 43.9%, and there was a 13.2 month PFS. NCCN guidelines recommend the use of selpercatinib for patients with RET fusion-positive CRC.

## 9. Deficient Mismatch Repair and Immunotherapy

Immunotherapy represents another breakthrough in personalized approaches to care for patients with mCRC. Several therapies have come onto the market for patients with deficient mismatch repair (dMMR) or high microsatellite instability (MSI-H) CRC. Checkpoint inhibition initially demonstrated promise in melanoma [85]. It has since been studied in many different malignancies, including CRC [86]. The most promising results of immunotherapy in mCRC have been with checkpoint inhibition for patients with dMMR/MSI-H CRC. Approximately 15% of all CRCs are dMMR/MSI-H, but the prevalence falls to about 7% in mCRC [87]. Multiple checkpoint inhibitors have demonstrated promising results for these patients.

Pembrolizumab is a humanized, monoclonal antibody and is a PD-1 inhibitor. It was initially studied in a phase II clinical trial for patients with mCRC [88]. Patients with dMMR/MSI-H and proficient mismatch repair (pMMR). The ORR was 40% for patients with dMMR/MSI-H and 0% for patients with pMMR. A subsequent multicenter, phase II trial evaluated pembrolizumab in patients with chemorefractory dMMR/MSI-H mCRC [89]. The ORR was 33%. The median duration of response in patients treated with ≥1 prior line of therapy was 4.1 months. A multicenter, phase III RCT (KEYNOTE-177) evaluated pembrolizumab versus chemotherapy with or without bevacizumab or cetuximab for dMMR/MSI-H mCRC [90]. The results of this trial noted improved PFS in the pembrolizumab group compared with the chemotherapy group at 16.5 versus 8.2 months, respectively. The ORR was also improved at 43.8% versus 33.1%. An updated analysis reported that the median overall survival was not reached in the pembrolizumab group versus 36.7 months in the chemotherapy group [91]. This finding was not considered statistically significant with *p* = 0.036 due to a prespecified α of 0.025. There was, however, significantly improved PFS and fewer treatment-related adverse events. 

Nivolumab is another humanized monoclonal antibody that targets PD-1. Initial trials evaluated the use of nivolumab as single-agent therapy [92]. Subsequent studies evaluated dual checkpoint inhibition with nivolumab and ipilimumab with more promising results [93]. Ipilimumab is a monoclonal antibody that targets CTLA-4. The combination was tested in the phase II CheckMate-142 trial in patients with dMMR/MSI-H mCRC with no prior treatment [94]. There was a 69% ORR, and median PFS and OS were not reached at the 24-month median follow-up. There were 13% of patients with a complete response. The phase III CheckMate 8HW study testing nivolumab/ipilimumab versus chemotherapy in the first-line setting is ongoing. The results of these trials have led to NCCN guideline recommendations for immunotherapy with checkpoint inhibition (ipilimumab/nivolumab or pembrolizumab) as first-line therapy for dMMR/MSI-H metastatic colorectal cancer.

Immunotherapy has been studied in combination with other therapies. Immunotherapy may be synergistic with anti-angiogenic therapy and immune checkpoint inhibition [95]. Preclinical studies have evaluated the relationship between angiogenesis and immune cell infiltration into the tumor microenvironment. These findings have led investigators to study immunotherapy in combination with VEGF inhibition in both patients with dMMR and pMMR. The multicenter, phase II AtezoTRIBE trial randomized patients with mCRC to FOLFOXIRI plus bevacizumab with or without atezolizumab [96]. The atezolizumab group experienced improved PFS at 13.1 versus 11.5 months with similar toxicity levels. The updated results of this trial demonstrated an improvement in OS in the atezolizumab group at 33.0 and 27.2 months [97]. Another multicenter, phase II trial evaluated the use of XELOX plus bevacizumab with or without adoptive cell immunotherapy for patients with mCRC [98]. The adoptive cell immunotherapy group had improved PFS at 14.8 versus 9.9 months. 

## 10. Ongoing Challenges and Future Directions

Despite a growing number of targeted therapies available for mCRC, the prognosis for patients with stage IV CRC remains poor [6]. Complete and/or durable responses to therapy are rare [99,100]. Patients with metastasis to the liver tend to have a worse prognosis than metastasis to other sites [101]. When targeting the EGFR pathway, downstream KRAS, NRSA, BRAF, and PIK3CA mutations decrease the response rate to therapy [102,103]. Similarly, anti-HER2 resistance can develop from downstream PIK3CA [104]. Efforts to target PIK3CA have not been fruitful [105]. Nevertheless, tumors with PIK3CA mutations are dependent on glutamine, and efforts to treat these patients with glutaminase inhibitors are underway [106]. Checkpoint kinase (CHK) is another promising target in the early stages of investigation and has demonstrated promise in preclinical studies, regardless of KRAS status, with early-phase clinical trials underway [107,108]. Fibrocytes and receptor tyrosine kinase c-Met have been implicated in the resistance to VEGF inhibitors, which represents another potentially actionable target [109,110]. Efforts to improve treatment response to immunotherapy in microsatellite stable colon cancer are also under investigation [111].

Strategies to overcome resistance to currently available regimens involve novel targeted drugs, multi-targeted therapies, and combination with immunotherapy. Scientists are discovering new pathways involved in tumorigenesis and resistance to targeted therapy, which offer new options for treatment. The evolution of the Consensus Molecular Subtype discussed above offers the potential to better understand individual tumor biology and determine the best treatment [112]. Another area of investigation is chimeric antigen receptor (CAR) T-cells. CAR T-cells are modified to recognize a tumor-specific antigen. While the use of CAR T-cells has promising results in hematological malignancies, no clinical trials have demonstrated efficacy in treating mCRC [113]. Cancer vaccines are also in early investigation, but clinical trial data are lacking [114]. The rising cost of cancer therapies presents a significant challenge to the field for all current treatments under investigation [115].

## 11. Conclusions

Molecular profiling of colorectal tumors is now the standard in CRC. Significant achievements have been made in investigating and developing targeted therapies for certain molecular profiles. Current therapy is more personalized than ever before. The EGFR, VEGF, HER2, and other pathways summarized above only scratch the surface of future therapeutic potential. Metastatic colorectal cancer continues to carry a poor prognosis. Continued investigation into new targets and new combinations of therapy is necessary to improve survival for these patients.

## Figures and Tables

**Table 1:** Targetable pathways in colorectal cancer.

Target	Mutation Prevalence	Therapy
EGFR	N/A	Cetuximab Panitumumab
BRAF V600E	8–12%	Encorafenib (with binimetinib)
RAS	50%	Sotorasib Adagrasib
VEGF	N/A	Bevacizumab Aflibercept Ramucirumab Regorafenib Fruquitinib
HER2	3–5%	Trastuzumab Pertuzumab Lapatinib Tucatinib Trastuzumab deruxtecan
NTRK	0.7%	Entrectinib Larotrectinib Repotrectinib
RET	0.2%	Selpercatinib
MSI-H/dMMR	15%	Pembrolizumab Nivolumab Ipilimumab

**Table 2:** Consensus molecular subtypes of colorectal cancer.

Subtype	Prevalence	Features
CMS1 (Microsatellite instability immune)	14%	Hypermutated Microsatellite unstable Strong immune activation
CMS2 (Canonical)	37%	Epithelial WNT and MYC signaling activation
CMS3 (Metabolic)	13%	Epithelial Metabolic dysregulation
CMS4 (Mesenchymal)	23%	TGF-β activation Stromal invasion Angiogenesis
